# Cholinergic Potentiation of Restoration of Visual Function after Optic Nerve Damage in Rats

**DOI:** 10.1155/2017/6928489

**Published:** 2017-08-27

**Authors:** Mira Chamoun, Elena G. Sergeeva, Petra Henrich-Noack, Shaobo Jia, Lisa Grigartzik, Jing Ma, Qing You, Frédéric Huppé-Gourgues, Bernhard A. Sabel, Elvire Vaucher

**Affiliations:** ^1^Laboratoire de Neurobiologie de la Cognition Visuelle, École d'optométrie, Université de Montréal, Montréal, QC, Canada; ^2^Institute of Medical Psychology, Otto von Guericke University, Magdeburg, Germany

## Abstract

Enhancing cortical plasticity and brain connectivity may improve residual vision following a visual impairment. Since acetylcholine plays an important role in attention and neuronal plasticity, we explored whether potentiation of the cholinergic transmission has an effect on the visual function restoration. To this end, we evaluated for 4 weeks the effect of the acetylcholinesterase inhibitor donepezil on brightness discrimination, visually evoked potentials, and visual cortex reactivity after a bilateral and partial optic nerve crush in adult rats. Donepezil administration enhanced brightness discrimination capacity after optic nerve crush compared to nontreated animals. The visually evoked activation of the primary visual cortex was not restored, as measured by evoked potentials, but the cortical neuronal activity measured by thallium autometallography was not significantly affected four weeks after the optic nerve crush. Altogether, the results suggest a role of the cholinergic system in postlesion cortical plasticity. This finding agrees with the view that restoration of visual function may involve mechanisms beyond the area of primary damage and opens a new perspective for improving visual rehabilitation in humans.

## 1. Introduction

The visual system has a substantial plasticity and a potential of partial spontaneous visual recovery both in animals [1–3] and in patients with visual system damage [4–6]. Moreover, clinical studies have shown that the residual vision subsequent to neural trauma or neuronal degeneration can be improved by training [7–9]. Several approaches have tried to enhance this visual recovery by potentiating cortical plasticity and sensory functioning. In this regard, stimulating central neuromodulatory systems that facilitate sensory responses and cortical plasticity is an interesting avenue.

There is growing evidence that the cholinergic system is involved in attention, cortical and synaptic plasticity, and potentiation of the cortical responses to visual stimulation. In rats, electrical or pharmacological stimulation of the cholinergic system when coupled with visual training enables long-term enhancement of responses in the visual cortex [10–13]. Particularly, acetylcholinesterase inhibitors (AChEIs), which prevent the degradation of the acetylcholine (ACh) and enable the buildup of ACh at the synapse, could be used for long-term enhancement of visual experience. AChEIs, such as donepezil (DPZ), are used for sustaining ACh action and treating the dementia symptoms in mild Alzheimer's disease [14]. Studies have shown that AChEIs enhance visual and cognitive capacities in both animals and humans [11–13, 15–18]. They also improve performance in behavioural tasks such as radial water maze, spatial recognition, and contrast sensitivity detection for healthy and hypocholinergic rats [18–21]. Moreover, combining cholinergic enhancement via DPZ administration with repetitive visual training increases the visual cortical responsiveness in healthy rats [13] and the learning in a perceptual-cognitive task in healthy young humans [17]. Therefore, administration of DPZ could be an effective method of cholinergic enhancement to potentiate the improvement of residual vision in visually deficient subjects.

The present study sought to evaluate whether cholinergic enhancement, by DPZ administration, can facilitate the restoration of the visual capacities following partial bilateral optic nerve crush (ONC) surgery in rats. Partial ONC is an effective model of diffuse axonal injury, which mimics visual impairment due to optic neuropathy or diffuse retinopathy. This model has been widely used to study neuronal damage, residual tissue left undamaged after the trauma or postinjury plasticity [22–24]. The spontaneous visual recovery after a mild ONC in rats has been characterized in behavioural studies, in terms of rate and extent. Usually, partial recovery (up to 20%) develops within three weeks postlesion [3, 25–28]. The visual capacity was thus measured behaviourally before and after partial ONC using brightness discrimination task (VIST) and physiologically by recording visual evoked potentials (VEPs). In addition, thallium autometallography was used to evaluate the neuronal activity via the ex-vivo quantification of visually induced cellular potassium uptake in the primary visual cortex (V1). The present result shows that the administration of DPZ in visually impaired rats induced a better recovery of brightness discrimination capacity in comparison to the non-DPZ treated animals. This result raises the possibility that DPZ may be used in processes of restoration of visual capacities.

## 2. Methods

### 2.1. Animal Preparation

**Figure 1:**
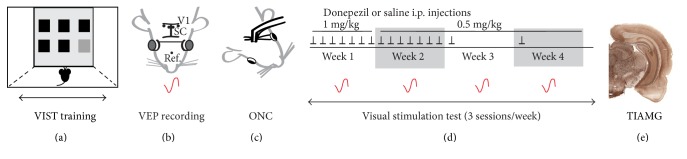
Experimental design. The study was performed over a period of 11 weeks: 5 weeks for the learning of the behavioural task training—VIST training, double-end arrow (a), 1 week for the baseline VEP recording (b) and the crush surgery (c), and 4 weeks for the repetitive behavioural and electrophysiological testing (d). Thallium autometallography (TlAMG) (e) was done one week after the last testing session. (a) The VIST test consisted of the detection of a bright stimulus (light gray square) among 6 openings (5 black and 1 gray square) on a touch screen. (b) When all rats reached an adequate brightness discrimination performance and success rate, electrodes (black dots) were implanted in the visual cortex (V1), the superior colliculus (SC), and in the nose bone (Ref.) in the brain/skull of the rats, together with a headstage to fix visual stimulation goggles (gray circles). A potential evoked by flash displayed through the goggles (VEP, red tracing) was recorded and set as a baseline. (c) Then animals were divided into three experimental groups: sham, ONC/DPZ, and ONC/saline, and bilateral partial optic nerve crush (ONC) or sham surgery was performed. (d) DPZ (1 mg/kg then 0.5 mg/kg) or saline i.p. injections were performed each day for the first 2 weeks post-ONC (black arrows) then once a week. VIST testing (3 times a week during 4 consecutive weeks) and the VEP recording (once a week during 4 consecutive weeks, red tracing) were performed. (e) One week after the end of the VIST testing and the jugular vein catheter implantation, the neuronal activity was measured by thallium autometallography (TlAMG).

A total of 20 adult Lister hooded rats (10 weeks of age when delivered to the animal facilities) were maintained in a 12 h light/dark cycle with ad libitum access to food and water during the adaptation period. Animals were handled for 10 minutes every day for 4 days before the beginning of the experiments. The rats were water deprived before the start of the behavioural study with limited access to water for 15 minutes per day because VIST learning was based on water reward. The study was performed over a period of 11 weeks (Figure 1). After VIST training, the baseline brightness discrimination capacity was acquired and the animals were randomly assigned to 3 groups: sham, *n* = 7 (sham-ONC and no injection), ONC/DPZ, *n* = 6 (ONC and DPZ injection), and ONC/saline, *n* = 7 (ONC and saline injection). Repetitive behavioural and electrophysiological testing and thallium autometallography were then performed. Note that some animals were excluded from VEP or TlAMG measurements because they did not fit the experimental criteria. All procedures were in accordance with ARVO Statement for the Use of Animals in Ophthalmic and Vision Research and with the guidelines of the Canadian Council for the Protection of Animals. All the procedures were accepted by the Ethics Committee of the Université de Montréal number 14-164 and of the IRB of the Landesverwaltungsamt Sachsen-Anhalt according to the German National Act.

### 2.2. Visual Stimulation Test

To quantify vision recovery, the visual stimulation test (VIST) technique [3] was used, where the visual performance of the rat was measured by the capacity of discriminating targets with different levels of brightness and the percentage of correct choices. 13 levels of brightness were displayed: 100% (white, level 1), 94%, 88%, 81%, 75%, 69%, 62%, 56%, 50%, 44%, 37%, 31%, and 25% (dark gray, level 13). Brightness discrimination was determined before ONC and after ONC during four consecutive weeks (3 sessions per week). Briefly, the water-deprived rats were placed in a Skinner box having six equally sized openings in the front panel attached to an infrared touch screen and a green light and water dispenser in the back panel. A trial consisted of illuminating one opening for 7 sec that the rat had to poke with its nose. After each correct choice, the rat was rewarded with a drop of water. For each trial, the target stimulus was randomly presented at a different location so that the rat had to track the position changes with repeated nose pokes until 4 consecutive correct choices were made, and then the next lower brightness level was presented. The task always started with the level 1 target then the level of brightness decreased. The performance was calculated by the following formula:

(1) $$\begin{array}{c} \%\;correct\;choices=\left(\frac{number\;of\;correct\;choices}{total}\right)\times 100. \end{array}$$

### 2.3. VEP Recording

#### 2.3.1. Headstage Implantation

In order to monitor VEPs at different time points, custom-made stainless steel Teflon-coated recording electrodes, 75 *μ*m uncoated diameter and 140 *μ*m coated with an impedance range of 100–500 kΩ (SS-3 T/HH; Science products GmbH), were chronically implanted in V1 (AP = −7 mm; ML = ±3 mm; DV = 0 mm) and in the superficial layer of the superior colliculus (Bregma coordinates: AP = −6.8; ML = ±1 mm; DV = 3 mm) under 75 mg/kg ketamine and 0.5 mg/kg medetomidine anesthesia according to Paxinos and Watson (1998). The reference electrode was implanted into the nasal bone, and an extra screw was implanted into the parietal bone to fix the headstage. Animals were allowed to recover during one week.

#### 2.3.2. VEP Recording

The VEPs were recorded on awaken rats once before the sham or ONC surgery for baseline value and once every week after the sham or ONC surgery. Before the first measurement and after a minimum of one-week recovery from stereotaxic surgery, rats were habituated to being handled and restrained in the electrophysiology set-up with opaque goggles fixed on the headstage. The homemade metal goggles displayed a 1300 mcd homogeneous illumination of the visual field to each eye individually by white 2.5 mm diameter light LED sources at 10 mm from the eyeball. To obtain VEP recordings, the pin sockets of the electrodes were connected with flexible cables to allow the rat to freely move its heads [29]. A preamplifier was connected to the headstage. Recording was performed in a dark room. Stimuli were delivered at 1 Hz for 2.5 min for one eye and then switched to the fellow eye after a 1 min break. The trigger signal was created using an isolated pulse stimulator (A-M Systems, USA) and was sent to the amplifier of the acquisition system as a marker. The signal was amplified ×20 and was band-pass filtered between 0.1 Hz–2 kHz using an 8-channel Porti system (Twente Medical Systems International B.V., Netherlands) and digitized with a 2 kHz sampling rate. The VEPs were analyzed in Matlab (Mathworks, Nattick, MA, USA) and EEGlab toolbox [30]. VEP amplitudes were measured by peak-to-peak analysis subtracting the signal of the contralateral nonstimulated eye. The final statistical analysis was performed only with the data of the rats that performed all the recordings during the 4-week post-ONC; some rats removed their headstage throughout the testing weeks and were therefore excluded from the final analysis (sham, *n* = 3; ONC/DPZ, *n* = 3; and ONC/saline, *n* = 6).

### 2.4. Optic Nerve Crush

A bilateral partial optic nerve crush was performed under ketamine (50 mg/kg, i.p.) and xylazine (10 mg/kg, i.p.) anesthesia [3, 22, 31]. The optic nerve was exposed from the lateral side of the eye and then crushed with calibrated forceps at a distance of 2-3 mm from the eye for 30 sec. Retinal blood supply and dura were left intact. An antibiotic eye ointment (Aureomycin; Lederle Arzneimittel GmbH, Wolfratshausen, Germany) was topically applied on both eyes after the surgery to prevent inflammation. In the sham group, the same surgery steps were made but the optic nerve was not crushed.

### 2.5. Drug Administration

DPZ (Sigma Aldrich, St-Louis, MO, USA) was prepared freshly in a sterile 0.9% NaCl solution and administered i.p. at the end of the day to avoid any acute effect. On the first week post-ONC, a loading dose of DPZ (1 mg/kg) was administered daily [12]. On the second week, a lower dose of DPZ (0.5 mg/kg) was administered daily [18]. We have previously shown that both doses strongly enhance the visual cortex reactivity assessed by VEP [13]. As DPZ is eliminated from the body by renal excretion and as urination was reduced due to the water restriction regime, a maintain dose was given on weeks 3 and 4 (0.5 mg/kg of DPZ, once a week). The same volume of saline, but without DPZ, was injected in the control animals.

### 2.6. Thallium Uptake

A thallium-chelate solution was administered 1 week after the end of the behavioural study and the implantation of the catheter, in a dark room during visual stimulation with a visual flickering in the left eye through the goggles to perform thallium autometallography (TlAMG). TlAMG is based on the bioaccumulation of thallium ions that substitute potassium ions and accumulate in cells and neurites during neuronal activation through ATPase channels. Thallium is then fixed by the perfusion of a sodium sulfide solution and is developed with silver for visualization under a microscope, as previously described [32, 33]. Briefly, catheters were implanted in the jugular vein. After 2-3 days postoperation, the catheter was connected to a polyethylene tube and 1 ml of a freshly prepared 0.05% thallium diethyldithiocarbamate solution in 0.9% NaCl was slowly injected over a period of 4 min during visual stimulation. After rinsing, 2 ml of sodium sulfide solution (0.32% Na2S in 100 mM phosphate buffer pH 7.4) and a sulfide glutaraldehyde solution (0.16% Na2S and 3% glutaraldehyde in 100 mM phosphate buffer pH 7.4) were bolus injected. The brains were then removed and immersed overnight in acrolein solution for fixation and then cryoprotected for 48 h in 30% sucrose in 0.1 M phosphate buffer, pH 7.4 at 4°C.

The brains were frozen and cut with a Leica cryostat into 25 *μ*m thick sections. Sections were air dried and treated with 0.1 N HCl to remove zinc sulfide. Then, sections were stained in a standard arabic gum developer used for autometallography [33, 34] for 150 min in the dark for the different groups (sham, *n* = 4; ONC/DPZ, *n* = 3; and ONC/Saline, *n* = 6); some rats were excluded because they removed their headstage, and some died after the catheter implantation.

Sections of interest containing V1 and subcortical structures were selected according to Paxinos and Watson (1998) and photographed with a Fuji FinePix S2 Pro digital camera mounted on a Leica DMR microscope. Photographs were displayed using the Adobe Photoshop software for Macintosh. The NIH image software (ImageJ for MacOS X) was used for the analysis of thallium uptake patterns. The coloured photomicrographs were converted to gray scale images using unweighted conversions in Adobe Photoshop. Gray levels were determined for each animal at the level of V1 and were compiled for analysis. High gray values correspond to low staining intensity, that is, low neuronal activity.

### 2.7. Statistical Analysis

Statistical analysis was performed using SPSS 17.0 (SPSS Inc., Chicago, IL, USA). In order to reveal the effect of the crush within each group, the brightness discrimination thresholds and percentage of correct choices before and after the ONC surgery were tested using pairwise *t*-test. Secondly, for the behavioural data, the brightness discrimination thresholds and percentage of correct choices were compared between the ONC groups for time and drug effects using mixed model ANOVA, with *p* < 0.05 being considered significant. For the VEP and optic density analysis, and given that the rat number was small, the between-group difference was evaluated using nonparametric Kruskal-Wallis test. This test was performed for each time point of the VEP measurement after ONC and for the TlAMG measurement performed at the end of the behavioural experiment. The Bonferroni correction was applied to compensate for multiple testing conditions, and post hoc pairwise comparisons were carried out with the significance level set at *p* < 0.05.

## 3. Results

### 3.1. Visual Stimulation Test

**Figure 2:**
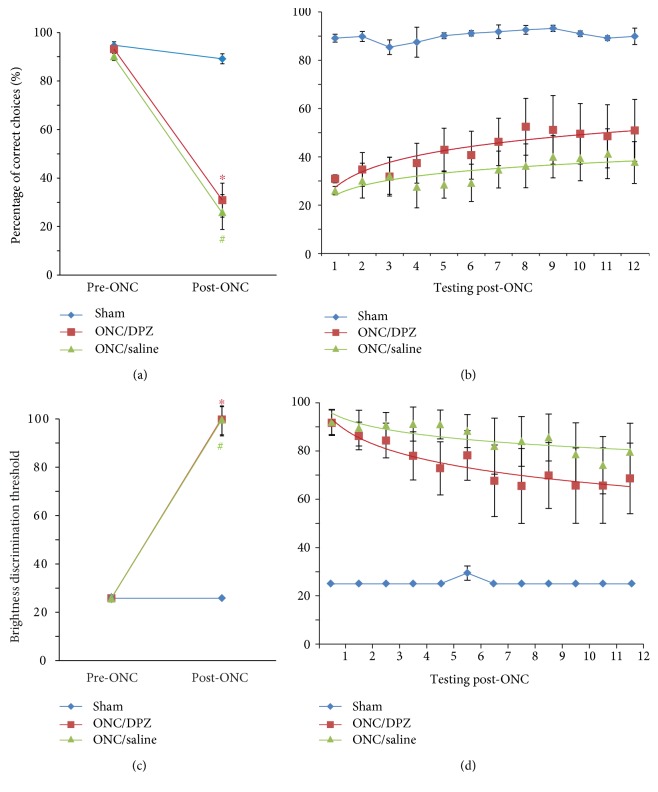
Brightness discrimination capacity and visual performance of the rats before and after the partial optic nerve crush or sham surgery. The visual performance evaluated by the VIST was characterized for the 3 groups: sham (blue), ONC/DPZ (red), and ONC/saline (green). Two parameters were calculated: the success rate (percentage of correct choices (a), (b)) and the brightness discrimination threshold (c), (d). VIST was performed before (pre-ONC) and after the ONC for 4 weeks. (a), (c) In comparison to the pre-ONC value, the ONC/saline and ONC/DPZ group percentage of correct choices (a) and brightness discrimination (c) showed a significant reduction after the ONC (^∗^*p* < 0.01, ONC/saline compared to pre-ONC; ^#^*p* < 0.01, ONC/saline compared to pre-ONC). (b), (d) Success rate and brightness discrimination were partially restored after the crush in both ONC/saline and ONC/DPZ groups, but the last group was performing better than ONC/saline group. Points in (b), (d) represent the testing session number (3 tests per week).

All the animals learned the brightness discrimination task easily and reached a discrimination performance of at least 25% of brightness before the ONC (Figure 2). The success rate (percentage of correct choices) was over 90% for all the animals (Figure 2(a)). These levels of discrimination performance and success rate were maintained in the sham group after the sham-ONC surgery and during the four weeks of VIST testing (Figures 2(b) and 2(d)). In contrast, both brightness discrimination threshold (ONC/DPZ: *t*_(5)_ = −12.649, *p* = 0.000; ONC/saline: *t*_(6)_ = −12.871, *p* = 0.000) and success rate (ONC/DPZ: *t*_(5)_ = 8.447, *p* = 0.000; ONC/saline: *t*_(6)_ = 8.578, *p* = 0.000) were significantly impaired by ONC in both ONC/DPZ and ONC/saline groups (62–67% reduction) (Figures 2(a) and 2(c)).

A mixed model ANOVA was further used to determine the effect of the time and drug through the 4 weeks of testing post-ONC in the ONC groups (Figures 2(b) and 2(d)). There was a main effect of time (brightness discrimination, *F*_(1, 11)_ = 5.990, *p* = 0.000; success rate, *F*_(1, 11)_ = 3.505, *p* = 0.000) and of drug (brightness discrimination, *F*_(1, 1)_ = 134.068, *p* = 0.000; success rate, *F*_(1, 1)_ = 26.868, *p* = 0.001). However, there was no interaction between time and drug (brightness discrimination, *F*_(1, 11)_ = 0.891, *p* = 0.551; success rate, *F*_(1, 11)_ = 1.138, *p* = 0.341).

### 3.2. Electrophysiology Results

**Figure 3:**
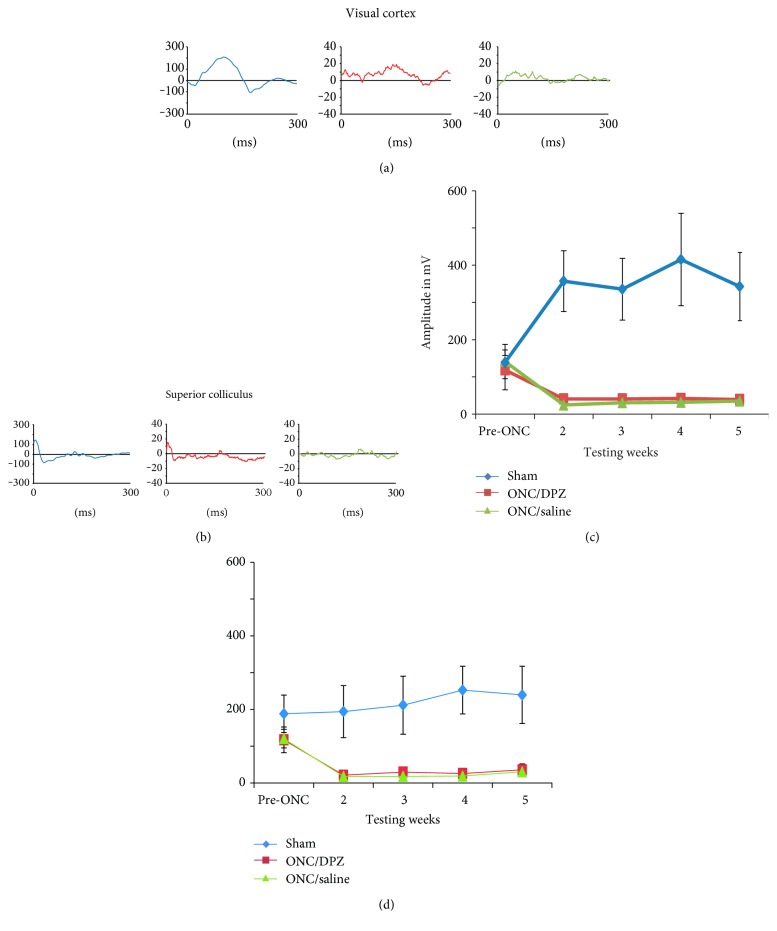
Visual evoked potentials recording in the primary visual cortex and the superior colliculus before and after the partial optic nerve crush. Amplitude of the VEP recorded before and once a week for four weeks after the ONC in the visual cortex (a), (c) and in the superior colliculus (b), (d) for the 3 groups: sham (blue), ONC/DPZ (red), and ONC/saline (green). (a), (b) VEP tracing examples recorded at week 5 in the visual cortex (a) and the superior colliculus (b). VEP amplitudes show a substantial drop in both ONC groups after the crush and a significant reduction of the VEP amplitude compared to sham groups both in the visual cortex (c) and in the superior colliculus (d). This indicates that cortical and subcortical visually evoked activity was not restored after the crush for any treated groups.

In order to evaluate whether the cholinergic treatment had an effect on the visual cortex and superior colliculus response after the crush, the visual evoked potentials were compared between the 3 groups: sham, ONC/DPZ, and ONC/saline (Figure 3). A substantial decrease of the amplitude of the VEPs was observed after the crush in both crush groups (ONC/DPZ V1 and SC ≈ 80 mV, ONC/saline V1 ≈ 100 mV, and SC ≈ 90 mV) in contrast to the VEPs in the sham group that remained high. VEP values were significantly decreased in the ONC compared to the sham groups (Kruskal-Wallis, testing week 5, V1, H (2,9) = 6.385, *p* = 0.041 and SC, J (2,9) = 6.231, *p* = 0.044). These results show that the electrophysiological response was not restored throughout the post-ONC period.

### 3.3. Thallium Autometallography (TlAMG)

**Figure 4:**
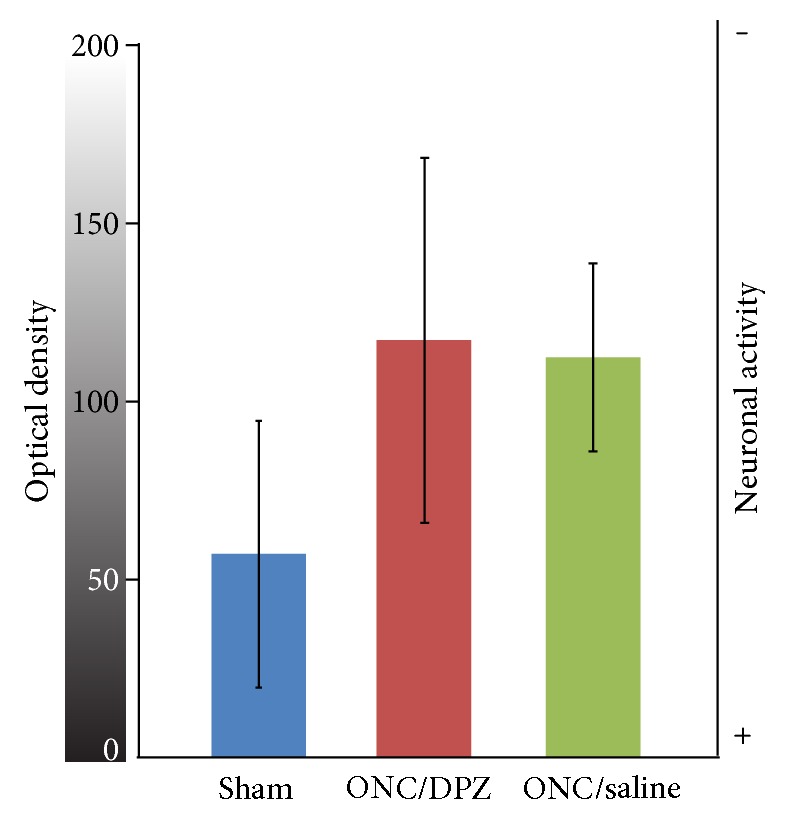
Cortical neuronal activity measured by thallium autometallography. Optical density (0 (black) to 255 (white) gray levels) was measured within V1 on coronal sections of the brain of rats having been perfused with a thallium solution during monocular flicker visual stimulation (see text). The high gray values correspond to low neuronal activity gradient (scale on the right). The optical density within the visual cortex was not significantly altered 5 weeks after the ONC, as shown for the ONC/DPZ (red) and ONC/saline (green) groups, compared to sham group (blue).

Neuronal activity was assessed 5 weeks postcrush in V1 using TIAMG (Figure 4). The optical density was measured on coronal sections, high gray levels corresponding to low staining intensity, that is, low neuronal activity. The statistical analysis between groups showed no significant difference of optical density between the 3 groups at this time point (Kruskal-Wallis, *H*_(2, 11)_ = 2,007, *p* = 0.375).

## 4. Discussion

We tested the effect of DPZ administration on the recovery of brightness discrimination in rats after a bilateral partial ONC. All ONC groups showed a significant impairment of brightness discrimination thresholds (reduction of 60% from initial value) after the crush, followed by a gradual restoration of brightness discrimination (up to 40% of the initial value in the ONC-DPZ group). The rats treated with DPZ had an overall better performance than the rats treated with saline. Both groups had spontaneous recovery of brightness discrimination and success rate during the 4 weeks of post-ONC testing. DPZ treatment did not improve V1 cortical activity measured by VEP after the ONC. Together, these results suggest that DPZ may help visual recovery by enhancing visual processing efficiency.

### 4.1. Optic Nerve Crush Induces an Impairment Followed by a Gradual Recovery of the Visual Capacities

In this study, we focused on the extent of brightness discrimination recovery following an ONC. All ONC groups had a significant drop of performance after the crush which was followed by a gradual but partial significant function recovery throughout the post-ONC period. Moreover, sham and both ONC groups showed similar TI^+^ uptake as evaluated 5 weeks post-ONC with optical density, indicative of a substantial neuronal activity in V1 in both groups. These results are in agreement with previous studies showing the same dynamics for both the behavioural VIST measurements [3] and thallium uptake 6 weeks after ONC [35]. However, when measured at different time points thereafter, TI^+^ uptake is first reduced in the dorsolateral geniculate nucleus and cortex after ONC but a normal cortical activity is restored 6 weeks after the crush [35]. Whether this cortical activity is related to visual function is not known. However, the amplitude of VEP evoked by visual stimulation is not restored at any time point after the ONC in V1 nor in the superior colliculus. This suggests that the visual pathways from the retina to the cortex and superior colliculus are still damaged and that the VEP is insensitive to any dynamic change that impacts the retina. Some recent studies show axonal regeneration following optic nerve crush in mice [36, 37]. However, the functional recovery in our animals occurred as early as on week three postinjury, which is well ahead of any possible axonal regrowth which could add to functional improvement [3, 29, 38–40]. Thus, on the one hand, there is a partial recovery of visual performance of the animals and neuronal activity in V1 but, on the other hand, no visually induced responses, suggesting that the behavioural recovery is rather due to plastic changes within cortical circuitry.

These results are in agreement with a structural-functional mismatch between behaviour and anatomical changes in the damaged optic nerve [41, 42]. It is possible that plasticity of neuronal activity takes place in V1. Several studies in the past have used elaborated optic nerve or retinal lesions to document cortical reorganization [43–47]. Following the loss of visual input, recovery of visual capacities are accompanied by plasticity of cortical circuits and cross-modal innervation in or near the lesioned area [48]. A strengthening of cortico-cortical connections is reported in animal models of retinal lesions to compensate for the loss of retinal input [49–51]. Furthermore, in humans with optic nerve damage, a recovery of their vision is observed following treatment with brain current stimulation, associated with a partial restoration of the network organization [52, 53]. These studies show that following visual system damage, both long-range lateral connections and large-scale functional connectivity networks are altered. This indicates that the remapping and the functional reorganization throughout the brain may be involved in vision recovery. Surviving ganglion cells, in combination with stronger cortico-cortical connections, can be considered to provide the mechanism of spontaneous recovery that occurs approximately within the 3 weeks post-ONC [41].

### 4.2. Donepezil Improves Visual Function following Optic Nerve Crush

Our finding that the ONC/DPZ group shows better brightness discrimination performance compared to the ONC/saline group gives the first evidence that the cholinergic system may be critical for the recovery process.

It has already been shown that the cholinergic system is involved in reinforcing the thalamocortical connections. For example, presynaptic nicotinic receptors on thalamocortical fibres boost thalamic input to the cortex [54] and more generally ACh strengthens the feedforward pathway therefore enhancing sensory performance [55–58]. Since the visual information arriving from the thalamocortical pathway to V1 is diminished by the ONC, the ACh should have an enhancing effect on the remaining thalamocortical connections, therefore facilitating the cortical responses of the residual cells. However, the VEPs did not improve in the visual cortex nor in the superior colliculus for the 4 weeks post-ONC in either group (DPZ or saline), suggesting that either VEPs or physiological activity in the remaining retina-visual cortex pathways is insensitive to the changes—although after ONC, the surviving ganglion cells adapt to the need of the visual functions and have a better activity [26, 59, 60]. In addition, donepezil could by itself have a protective effect of ganglion cells in the retina [61, 62] but the VEP recordings from the present study show that the retino-cortical function was not restored after 21 days after the crush. These results are in line with other studies using the ONC model in animals where the VEPs are deteriorated immediately after the crush and do not show any recovery for weeks [63, 64]. Additionally, in the case of optic disease in human, which is mimicked by the rat ONC model and which leads to partial vision loss, electrophysiology and imaging studies prove that diminished visual brain function related to the optic neuritis eyes is correlated with the extent of the optic nerve damage [65–67]. Since the VEPs did not improve after ONC in the ONC/saline nor in ONC/DPZ groups, it suggests that the expected cholinergic enhancement of thalamocortical input is not efficient enough to sustain the behavioural better performance of the rats. Whether the residual thalamocortical input is too weak to play a role in recovery or whether cholinergic system is not able to potentiate enough the remaining thalamic responses to induce changes in VEP needs to be determined by future studies.

The lack of effect of the DPZ treatment on VEP response together with the significant behavioural effect suggests a role of the cholinergic system in cortical plasticity [68]. In healthy rats, the role of the cholinergic system in visual enhancement has been reported using behavioural and electrophysiological studies [11, 13, 16, 69, 70]. Precisely, by combining visual exposure with electrical stimulation of the cholinergic system, rats showed an improvement of visual acuity in a water maze and the potentiation of the visual cortical responsiveness. Moreover, exposing rats to 2 weeks of visual stimuli coupled with DPZ administration enhances the visual cortical response [13]. Additionally, administration of cholinergic enhancers significantly improves behavioural performance in a visual task and contrast detectability in the healthy rats [12, 18]. In our study, DPZ induced better performance in the behavioural task, suggesting that this effect of cholinergic enhancement on cortical plasticity is also observed in our model of visual impairment.

The improved recovery of visual performance in ONC/DPZ group might also be due to the role of the cholinergic system in attention. ACh is shown to affect the strength of the connections in the visual cortex and enhance the relevant stimuli by facilitating glutamatergic feedback [71]. In fact, an enhancement of cholinergic concentration in the cortex promotes attention [72–74]. Furthermore, studies show that attentional cueing tasks improve vision restoration on patients with visual field loss therefore facilitating the visual perception recovery [75, 76]. Additionally, cholinergic enhancement induces potentiation in the cortical responsiveness regardless of the type of stimuli [77, 78]. Previous studies in healthy animals show that cholinergic enhancement potentiates visual cortical response and visual performance [10, 13, 18, 21, 69, 70]. In human studies, enhancing the cholinergic system with AChEIs improves the performance in visual and behavioural tasks that require attention [79–83], suggesting an improvement of efficiency of the visual processing. In our study, DPZ allowed better detection of relevant stimuli after the crush, which could result from increased attention capacity. Therefore, the implication of the cholinergic system in attentional processes might have affected visual recovery post-ONC.

## 5. Conclusion

In summary, we showed that cholinergic enhancement induces better visual recovery following an optic nerve crush. This agrees with the proposal that ACh enhancement can potentiate spontaneous visual recovery by reinforcing plasticity and attentional processes.
